# Effects of a wrapping closure lacing system on wearing comfort, lock-in stability, and lower-limb muscle demand during prolonged running

**DOI:** 10.3389/fspor.2026.1775046

**Published:** 2026-03-16

**Authors:** Yichen Wang, Wei Huang, Nan Zhang, Tony Lin-Wei Chen, Ming Zhang

**Affiliations:** 1 Department of Biomedical Engineering, Faculty of Engineering, The Hong Kong Polytechnic University, Hong Kong, China; 2 Shenzhen Icomwell Intelligent Medical Technology Co., Ltd., Shenzhen, Guangdong, China; 3 Research Institute for Sports and Technology, The Hong Kong Polytechnic University, Hong Kong, China

**Keywords:** footwear lacing, lock-in stability, muscle electrophysiology, wearing comfort, wrapping closure

## Abstract

**Introduction:**

Wrapping closure systems are alternative footwear lacing methods that could influence perceived fit and movement biomechanics, yet their performance during prolonged running and impacts on muscle activities remain underexplored. This study compared the effects of a wrapping closure system (dial-based lacing system, DLS) and conventional laces (CL) on wearing comfort, lock-in stability, running kinematics, and lower-limb muscle activation in trained marathoners during an extended running session.

**Methods:**

Twenty marathoners completed two 50-minute treadmill running trials on separate days using either DLS or CL. Wearing comfort was indicated by donning/doffing time and repeated VAS ratings. Lock-in stability was evaluated via dorsal foot pressure measures, shoe-throat width, and incidence of lace loosening. Running kinematics were assessed by motion capture and musculoskeletal modeling. Surface EMG was recorded from four lower-leg muscles. Between-group differences were examined statistically.

**Results:**

DLS produced shorter donning/doffing time and lower discomfort ratings during the mid-to-late running stage compared to CL (*p* < 0.025). There was no incidence of lace loosening in DLS while that of CL was 10% (*p* = 0.500). Pressure fluctuations and peak tibialis anterior activation were significantly smaller in DLS than CL (*p* < 0.036) at several time checkpoints of the running trials. No significant differences were observed in other kinematic and EMG measures.

**Conclusion:**

DLS demonstrated greater wearing comfort than CL. DLS also reduced variability in dorsal foot pressure and lowered local muscular demand without altering overall running kinematics, which indicates the potential advantage of DLS in maintaining foot–shoe coupling during endurance running.

## 1 Introduction

Running is one of the most accessible and widely practiced forms of physical activity worldwide and is frequently promoted for various health benefits (1). As participation in running-related events continues to grow, interest has also increased in optimizing footwear to enhance performance while minimizing injury risks. Contemporary running shoes are complex systems shaped by both design features and manufacturing procedures, in which midsole cushioning, heel-to-toe geometry, outsole configuration, and upper construction interplay to influence lower-limb biomechanics and perceived comfort (2–4). Footwear design can therefore modulate how runners interact with the ground, affecting foot-shoe coupling and gait patterns, with downstream implications for running economy and overuse injury risk (5, 6). Within this context, the interface between the foot and the shoe upper—where fitting, stability, and in-shoe pressure distribution can be governed—is increasingly recognized as an important but comparatively understudied determinant of both performance and comfort (4, 7).

Among the many features of running footwear, the lacing or closure system plays a central role in regulating fitting by adjusting upper tightness, foot containment, and foot–shoe coupling (4, 8). Research has shown that altering lacing tightness or pattern can change plantar and dorsal pressure distributions, in-shoe displacement, and perceptions of stability. For example, tightening conventional laces reduces rearfoot motion and heel slippage but increases localized pressures over the instep and forefoot, whereas looser lacing increases pressure-time integrals under the toes and is associated with greater perceived in-shoe movement (8, 9). More recently, an alternative wrapping closure system has been introduced to provide a more even distribution of tension across the upper and allow quick, repeatable adjustments of the tightness. Compared to standard laces, a wrapping closure system was reported to improve agility-task performance, reduce peak subtalar eversion velocity, and lower dorsal/plantar rearfoot pressures in sports footwear while also improving tactile sensation of the wearers (7, 10–13). In parallel, observational and review work suggests that individualized lacing techniques can be used strategically to enhance comfort and potentially reduce injury risk in specific populations (4, 8, 14). Taken together, these studies indicate that closure systems and lacing strategies are functionally important design variables that can influence both movement biomechanics and wearing perceptions.

Despite these advancements, several important research gaps remain in the literature. First, most existing studies adopted relatively short, task-based protocols (e.g., agility drills and short trail segments), providing only a snapshot of how different lacing systems affect comfort and kinematics under immediate conditions (4, 8, 14). It is plausible that differences in runners' responses to various lacing systems can be amplified during prolonged running—when highly repetitive exercises may alter both the interaction at the foot–shoe interface and the perception of lockdown over time—though supportive evidence was lacking. Moreover, a body of work in related areas focused on gait adaptations and lower limb loading redistribution under different lacing conditions, yet limited attention has been given to changes in muscle electrophysiology. Such lower-leg muscle activation patterns may reflect compensatory motor strategies for maintaining in-shoe motion and foot–shoe stability when lacing methods are altered. Lastly, prior studies that mapped the pressure at the foot-shoe interface normally report the peak or average pressure reading to indicate the degree of foot-shoe coupling (15). These metrics, however, may not capture the pressure oscillation caused by small foot-shoe relative movements and intermittent unstable fitting from stride to stride. We therefore proposed a supplementary variable—pressure fluctuation, defined as the drop between peak and volley pressure values within a gait cycle, to reflect the temporal consistency of foot-shoe coupling. The average pressure fluctuation across consecutive steps was expected to provide better insight into the dynamic lock-in stability during prolonged running and relate closely to comfort perception and the compensatory muscle activation for maintaining foot-shoe coupling.

In light of the abovementioned scenarios, the current study aimed to compare a wrapping closure system with conventional laces in trained marathon runners during 50-minute treadmill runs, with the aim of quantifying their effects on wearing comfort, lock-in stability (including lace loosening and dorsal-foot pressure characteristics), running kinematics, and lower-limb muscle activation.

## 2 Methodology

### 2.1 Participants

Due to the limited availability of published data with a similar study design for reference, we performed the sample size estimation based on a pilot trial comprising the first five participants in the cohort. Since our study mainly focused on between-lacing differences, we simplified the sample planning to a subject-level paired comparison by collapsing all time measures within each lacing condition. Using a two-sided paired t-test framework with *α* = 0.05 and *β* = 0.2, the calculation reported a minimum sample size of 15. To ensure adequate power in the presence of potential variability and attrition, we eventually recruited 20 participants (10 males: 172.30 ± 4.42 cm, mass: 63.60 ± 8.87 kg, age: 40.90 ± 5.61 yrs and 10 females: 157.85 ± 5.04 cm, mass: 51.40 ± 6.48 kg, age: 38.10 ± 7.67 yrs). Inclusion criteria were: aged 18–50 years, running at least 24 km per week for the past three years, meeting respective minimum requirements of marathon performance (males: full marathon finish time <4 h: females: full marathon finish time <4 h 45 min for at least one race during the past one year before the experiment), injury-free with no chronic cardiopulmonary diseases or musculoskeletal injuries that would interfere the running gait at the time of the experiments. All participants were fully informed of the experimental procedure and properly consented. They were also asked to fill out a questionnaire that collected information regarding their basic anthropometrics, race history, and training regimen. The research protocol was approved by the Institutional Review Board (reference number: HSEARS20241027001).

### 2.2 Experimental procedure

**Figure 1:**
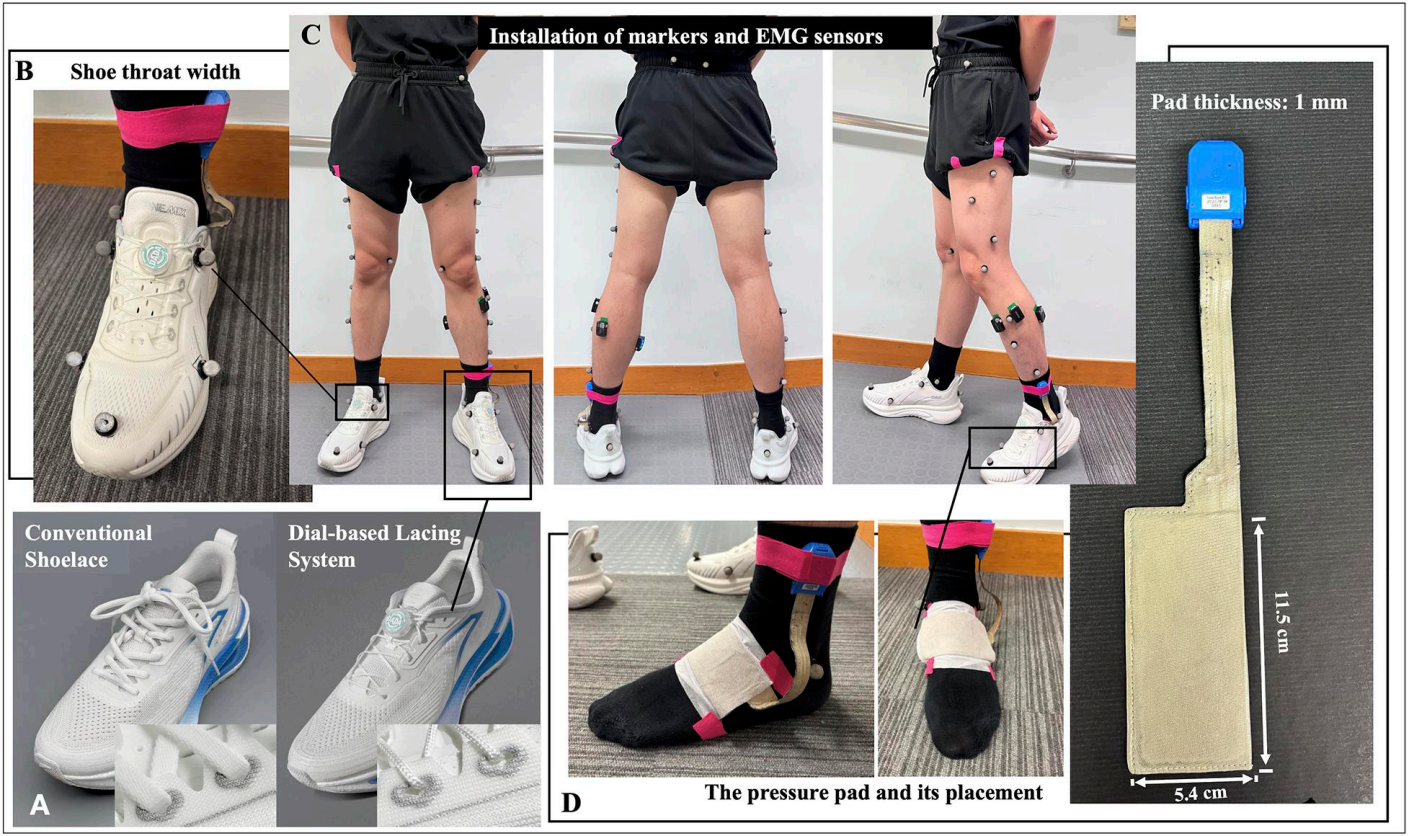
Experimental setups of the running trials. **(A)** the two lacing conditions and running shoes utilized in the current study; **(B)** markers on the eyelets to track changes in the shoe throat width; **(C)** installation of markers and EMG sensors; **(D)** specifications of the pressure pad and its placement on the dorsal foot.

A within-subject, repeated-measures crossover design with washout was used to compare the performance of two lacing systems (Figure 1A)—a wrapping closure system and the conventional laces (CL, the original polyester-based laces that came with the shoes). The wrapping closure system was represented by a dial-based lacing system (DLS, the DADA Dial System, Icomwell Ltd., Shenzhen, China). Each participant completed two treadmill running sessions on separate days, with a minimum washout period of 48 h between sessions to avoid fatigue bias and carry-over effects. The order of lacing conditions being tested was counterbalanced across participants.

The participants were assigned the same model of neutral running shoes (anonymous, Figure 1A). The original shoelaces were standard flat polyester laces that were tied using a standard bow knot. For the spin-lack condition, the shoelaces were replaced by a reel anchored at the tongue with a nylon-coated steel wire looping through eyelets. The fitting procedure required runners to start with a sitting position, lace the shoes, and adjust the tightness until self-reported optimal comfort was achieved. We recorded the time used to lace up (donning) for both lacing conditions using a digital stopwatch.

To track the running gait kinematics, a total of 39 retroreflective markers were attached to anatomical landmarks based on the configuration requirements of OpenSim 2392 (Opensim v4.5, Stanford University, Stanford, CA, USA) lower-limb model (e.g., anterior superior iliac spine, posterior superior iliac spine, lateral epicondyle, lateral malleolus, first and fifth metatarsal heads) (16). Two additional markers were placed at eyelets on both sides at the instep level to facilitate calculating the changes in the width of the shoes' tongue throat (Figure 1B). Marker trajectories were acquired at 250 Hz using an 8-camera motion capture system (Vicon Vantage v8, Vicon Motion Systems Ltd, Oxford, UK). Surface EMG was recorded from the tibialis anterior, peroneus longus, medial gastrocnemius, and lateral gastrocnemius using wireless sensors (Delsys Trigno, Delsys Inc., Boston, MA, USA) sampling at 1,000 Hz (Figure 1C). Skin preparation and sensor installation followed the SENIAM guideline (17). The two sensing systems were synchronized using a shared transistor–transistor logic pulse. In addition to these measures, a flexible wireless pressure pad (Novel loadpad, Novel, Munich, Germany) was inserted between the dorsal foot and shoe lining (Figure 1D). We applied Kinesio tapes (Kinesio, Albuquerque, NM, USA) on the edges of the pad to minimize its movement relative to the dorsal foot. The pad was placed in a fashion that its central line was perpendicular to the longitudinal foot axis and the foot instep sat at the center of the pad's sensing area (Figure 1D). The sensing area was connected to a control module affixed to the lateral malleolus using an adjustable strap and gauged the total dorsal pressure at the sampling rate of 120 Hz throughout the running trial.

Participants were given ample time to warm up before the trials and then completed the running sessions on a treadmill (RehaWalk, Zebris Medical GmbH, Baden-Württemberg, Germany) at a constant speed—12 km/h for males and 10 km/h for females. These speeds corresponded to sub-elite marathon performance levels for each sex group based on established criteria (18). We applied these speeds that slightly exceeded our participants' capacities to ensure a sufficient level of physical demand in the running trials and increase the likelihood of eliciting time effects (repeated measures) on the outcome variables. Each trial lasted 50 min. At 10-minute intervals (i.e., at the 10th, 20th, 30th, 40th, and 50th minutes), participants verbally reported their overall comfort using a 10-cm visual analogue scale (VAS), where 1 indicated “most comfortable” and 10 indicated “least comfortable.” Additionally, the number of spontaneous lace releases (i.e., loosening or unlocking events) was recorded throughout the trial.

Upon completion of the running trials, participants returned to a seated position and unlaced the shoes (doffing), for which the time consumed was recorded. Participants then completed a structured questionnaire identifying the top three factors influencing their comfort rating during the trial. They are suggested to choose three out of a list of predefined options: (1) containment, (2) flexibility, (3) stability, (4) breathability, (5) lightweight, (6) traction, and (7) “other”. On a separate setting on a non-running-trial day, we invited the participants to repeat the donning procedure and then increasingly tighten the lacing to “uncomfortable” and “painful” levels, at which points we recorded the corresponding pressure values for reference purposes.

### 2.3 Data processing and outcome variables

The kinematics data collected by motion capture analyses were low-pass Butterworth filtered at 8 Hz. A subject-scaled 2392 model in the OpenSim platform processed the data via inverse kinematics to reproduce the joint angles and segment orientation. Both measures were output using the model default reference frames. The EMG data were first band-pass filtered between 10 and 500 Hz using a fourth-order Butterworth filter to remove movement artifacts and high-frequency noise. A 50 Hz notch filter was then applied to eliminate powerline interference. The filtered data were smoothed using a 100-ms moving RMS window to obtain the linear envelope. The resulting linear envelopes were amplitude-normalized to the maximal EMG value of each muscle obtained from all running trials. This task-specific normalization approach is suitable for within-subject comparisons under the same movement conditions, whereby changes in the outcome measures can be attributed to different lacing systems rather than inter-individual scaling (19).

The outcome variables of this study were grouped into three categories. The first category is wearing comfort, which was represented by the time required for shoe donning/doffing, VAS scores of perceived comfort during running, and selection of factors contributing to comfort rating. Factor selection was quantified as the percentage of participants in the pool who selected a specific factor. The second category is lock-in stability, encompassing measures of in-shoe dorsal foot pressures (peak, valley, and fluctuation of the pressure values), changes in the width of the shoes' throat, and incidence of lace loosening during running. The dorsal foot pressure fluctuation was defined as the difference between the peak and valley values within a gait cycle. The third category—running kinematics and muscle EMG. Kinematic measures included the peak joint angles (e.g., knee flexion, ankle dorsiflexion/plantarflexion, and ankle internal/external rotations), spatiotemporal metrics (stride length and cadence), lower leg frontal-plane alignment (rearfoot pronation), peak foot-ground clearance, and intersegment coordination variabilities of coupled lower-leg segments. The knee joint has only one degree of freedom in model 2392 and therefore knee flexion (positive) is defined as the rotation of the tibia about the mediolateral axis in the femur knee frame (20). The ankle motion is represented by two coordinates: ankle dorsiflexion/plantarflexion is the talar motion about the mediolateral axis of the tibia frame (positive = plantarflexion) and ankle inversion/eversion is defined as the rotation of the rearfoot about a predefined oblique subtalar axis (positive = inversion). For spatiotemporal metrics, cadence was calculated from consecutive heel-strike events and expressed in steps per minute. Stride length was defined as the anteroposterior displacement between two consecutive heel strikes of the same foot. Rearfoot pronation was defined as the angle between the longitudinal axes of the tibia and rearfoot on the frontal plane. The foot-ground clearance and coordination variabilities were calculated using our published protocols (17, 21, 22). Briefly, the foot-ground clearance is the maximum vertical distance between the shoe's bottom and the ground surface. Coordination variability is the stride-to-stride variability (circular standard deviation) of inter-segment coupling angles quantified using a modified vector coding approach. Dorsal foot pressure metrics were reported at 5-minute intervals, whereas running kinematic and EMG variables were reported at 10-minute intervals to reduce data granularity and computational demand. All kinematic and EMG variables, except for the foot-ground clearance, were derived from the stance phase of the left limb. For each 10-minute interval, the data of all complete gait cycles from the final 1 min were analyzed. Peak values of the outcome variables were averaged across all available steps to obtain a representative value for each time interval.

### 2.4 Statistics

Normality of the data was assessed and confirmed by the Shapiro–Wilk test (W = 0.86–0.96, *p* = 0.007–0.566). Since the normality assumptions was satisfied, donning/doffing time was compared between the two lacing conditions using paired Student's t-tests. The between-group difference in the incidence of lace loosening was examined by the exact McNemar's test due to the small number of positive cases. A generalized estimating equation (GEE) model was employed to examine the interactions and main effects of lace condition and running time (both are within-subject factors) on variables repeatedly measured during running (e.g., VAS scores, pressure measures, kinematic, and EMG variables). The GEE model also incorporated covariates such as age, gender, running speed, and running experience to account for potential confounding effects. When a significant interaction between lacing condition and running time was identified, stratified GEE analyses were performed to isolate the effects of lacing condition at each fixed time checkpoint. Cohen's *d* and standardized estimated marginal differences (SEMD) were reported to indicate the effect sizes for significant paired Student's t-tests and GEE comparisons respectively. SEMD was calculated by dividing the GEE-estimated marginal mean difference by the square root of the model scale parameter. The effect size was interpreted as negligible (<0.20), small (0.20–0.49), medium (0.50–0.79), and large (≥0.80) based on Cohen's convention (23). All statistical analyses were performed using SPSS (v21, IBM Corp., Armonk, NY, USA) and significance was set at a global alpha level of *p* < 0.05.

## 3 Results

### 3.1 Wearing comfort

**Figure 2:**
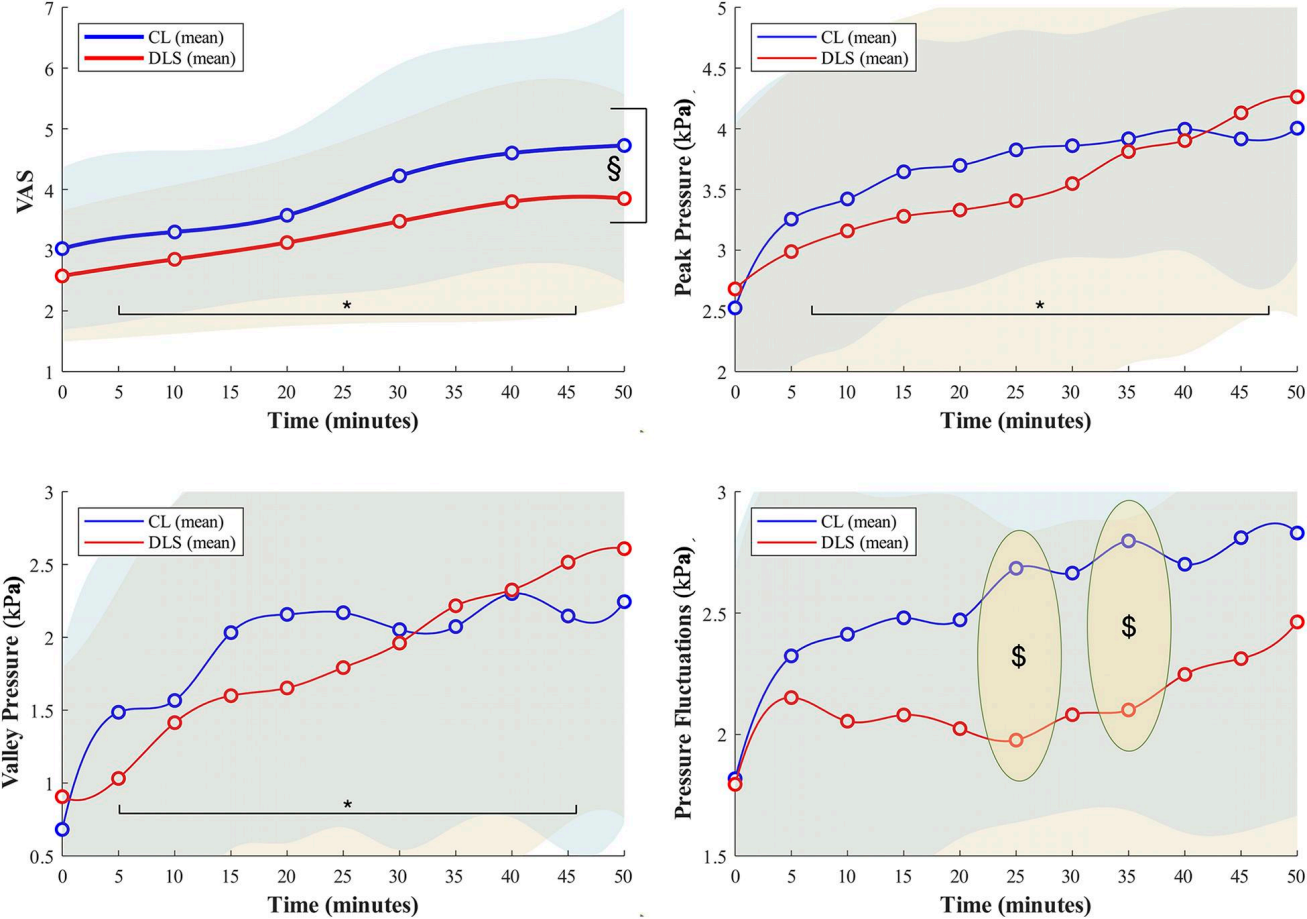
Changes in comfort ratings (VAS) and dorsal foot pressure measures as a function of time during the running trials. * indicates significant main effects of running time on the variables. § indicates significant main effects of lacing condition on the variable. $ indicates significant simple main effects of lacing condition on the variable.

The average donning/doffing time were 13.32s/8.23s in DLS, both of which were significantly lower than those (36.95s/17.26s) of CL (*p* < 0.001, Cohen'*d* = 0.73–1.57). GEE reported no interaction effects of lace condition ×  running time on overall wearing comfort. Analyses of the main effects of respective factors revealed that comfort rating gradually reduced (VAS of discomfort increased) as the running time increased (Wald *χ*^2^ = 19.27, *p* < 0.001, SEMD: 0.75). Every 10-minute increment in the running time caused the VAS to increase by 0.30 (Wald *χ*^2^ = 10.34–15.65, *p* < 0.001). Despite that, VAS in DLS was 0.67 lower than that of CL (Wald *χ*^2^ = 5.02, *p* = 0.025, SEMD: 0.41) across all time checkpoints, indicating an overall better wearing comfort in DLS compared to CL during the running trials (Figure 2). No significant covariances were found for this metric (Wald *χ*^2^ = 0.183–0.671, *p* = 0.180–1.773). “Containment” and “stability” were both selected by 70% of the participants as the dominant contributors to their ratings on the overall wearing comfort during running.

### 3.2 Lock-in stability

No incidences of lace loosening were observed with DLS during the running trials in all participants, whereas two cases of lace loosening were recorded with CL, resulting in an overall loosening rate of 10% for CL (*p* = 0.500). GEE reports no interaction and no main effects of the two primary factors on measures of the width of the shoe throat (Wald *χ*^2^ = 1.36–2.23, *p* = 0.244—0.693). The averaged dorsal foot pressure after comfort fitting was reached was 1.81 ± 0.92 kPa (0.49–3.89 kPa) across the two lace conditions. The value reached 134.02 ± 51.05 kPa and 201.74 ± 47.84 kPa at the “uncomfortable” and “painful” levels respectively. We found that the dorsal foot pressure fluctuated within a gait cycle, normally peaking at mid-stance and dropping to the lowest during the swing phase. GEE indicated no significant main effects of lace conditions (Wald *χ*^2^ = 0.00–1.27, *p* = 0.261–0.953) on peak and valley dorsal foot pressures within a gait cycle. Every 10-minute running time accumulated was associated with an increment of 0.31 kPa and 0.33 kPa in peak dorsal and valley dorsal foot pressures respectively (Wald *χ*^2^ = 39.75–86.27, *p* < 0.001). A significant lace condition × running time interaction was found on pressure fluctuations. The follow-up stratified GGE analysis showed that pressure fluctuations were 0.71 kPa (SEMD: 0.72) and 0.70 kPa (SEMD: 0.73) higher in CL than DLS at the 25th and 35th minute of the running trials respectively (Wald *χ*^2^ = 4.40–4.71, *p* = 0.030–0.036) (Figure 2). No significant covariances confounded the effects of the two primary factors on all pressure measures (Wald *χ*^2^ = 0.001–3.283, *p* = 0.070–0.990).

### 3.3 Running kinematics and muscle electrophysiology

**Figure 3:**
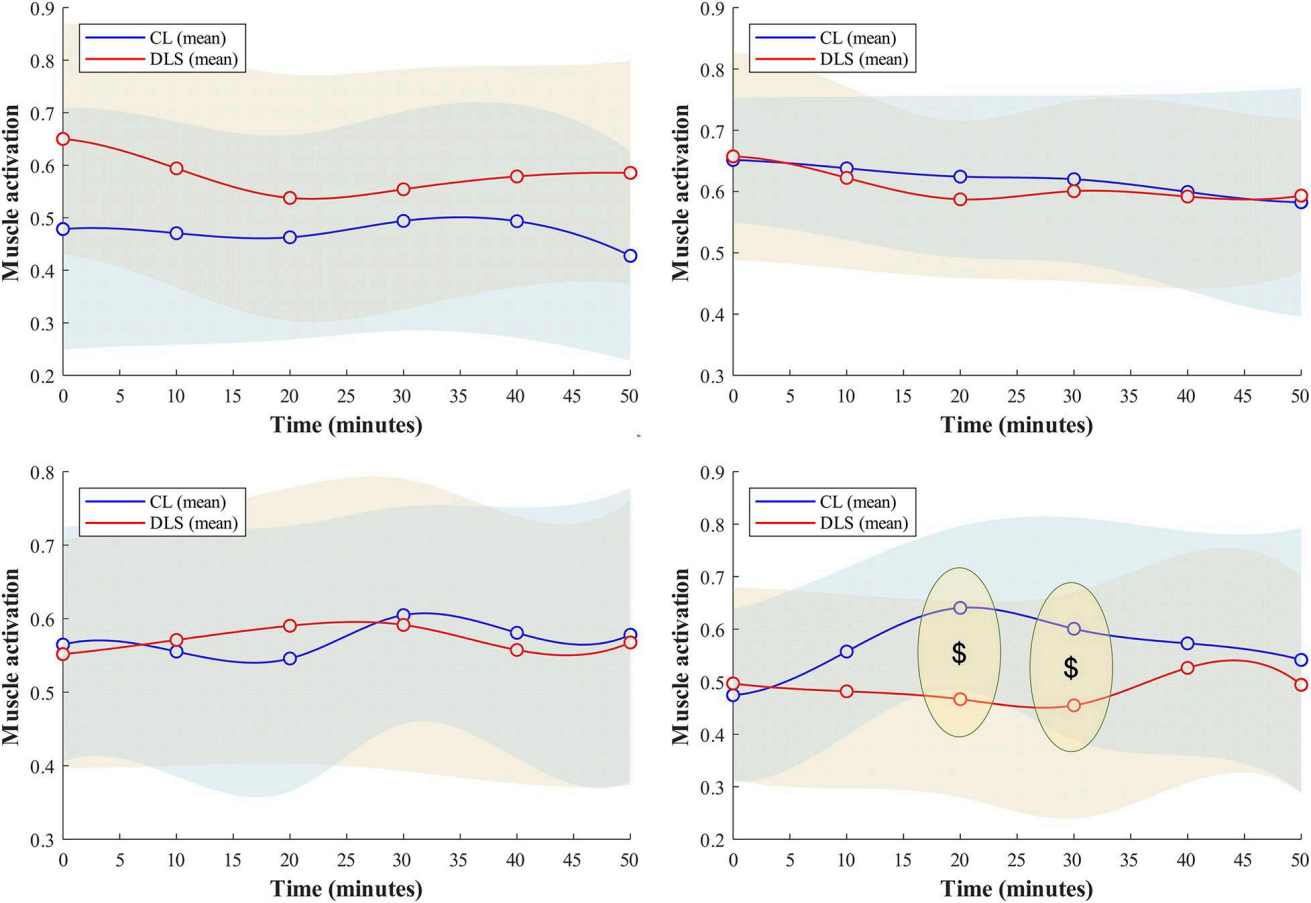
Changes in selected lower limb muscle activations as a function of time during the running trials. $ indicates significant simple main effects of lacing condition on the variable.

GEE reported a significant interaction between lace condition and running time on the peak EMG value of the tibialis anterior (Wald *χ*^2^ = 14.77, *p* = 0.005). Stratified GEE at a fixed level of the running time revealed that the peak tibialis anterior activation was significantly lower in DLS compared to CL at the 20th (SEMD: 0.99) and 30th (SEMD: 0.70) minutes of the running trials (Wald *χ*^2^ = 5.94–15.38, *p* < 0.015) after adjusting for covariances (Figure 3). Height was a significant confounder for this metric (Wald *χ*^2^ = 4.12, *p* = 0.042).

**Figure 4:**
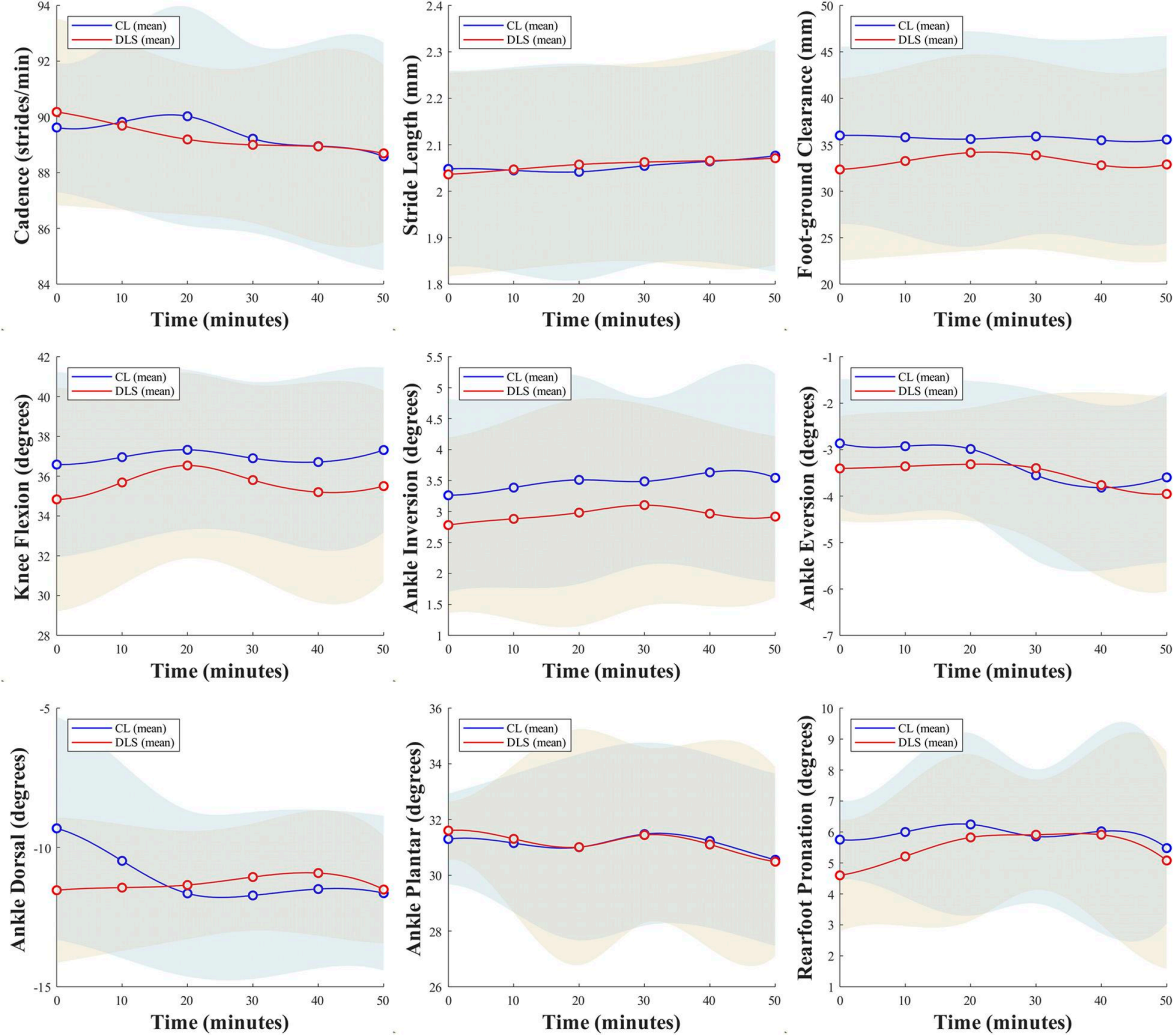
Changes in spatiotemporal parameters, peak joint angles, and lower limb alignment as a function of time during the running trials.

**Figure 5:**
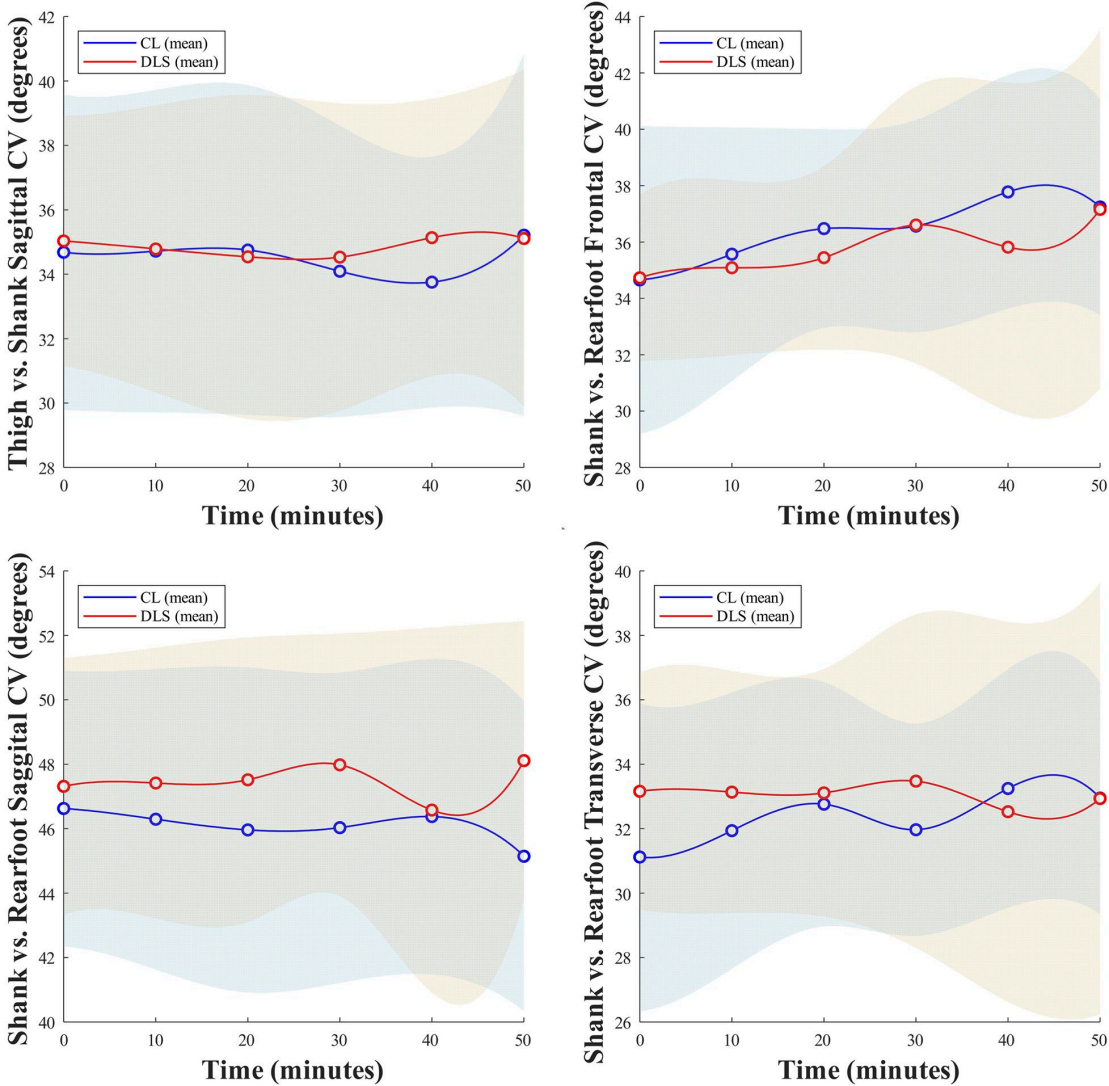
Changes in coordination variabilities of coupled lower limb segments as a function of time during the running trials.

No significant interactions of lace condition × running time nor main effects of the two primary factors were identified on peak activation of other included muscles, spatiotemporal parameters, peak joint angles, lower limb alignment, and intersegment coordination variabilities (Figures 4, 5).

## 4 Discussion

In this study, we evaluated and compared the effects of two lacing systems on wearing comfort, lock-in stability, running kinematics, and lower limb muscle activities. Compared to CL, DLS demonstrated superior wearing comfort by producing significantly shorter donning/doffing times and better VAS during mid-to-late stages of the running trials, despite that the comfort ratings gradually declined with accumulated running time in both lacing conditions. No lace loosening occurred in the DLS condition, whereas CL showed a total of 10% loosening incidence during the running trials. The peak and valley dorsal foot pressures increased over time in both lacing systems while pressure fluctuations within a gait cycle were significantly smaller in DLS than in CL at the midpoint of the running trials. EMG measures indicated a lower peak activation of the tibialis anterior in DLS compared to CL as the running time amassed over 20 min. No significant differences in EMG of other muscles and all metrics of running kinematics were observed between the two lacing conditions.

Our findings on comfort perception align well with those of Honert et al. (11, 12), who reported that runners consistently rated wrapping closure systems as providing superior fit and overall comfort compared to conventional lacing. In line with their work, participants in the current study also reflected that they perceived greater confidence and stride-to-stride consistency when using DLS (11), which afterward manifested as ranking “stability” and “lockdown” as the dominant contributors to the overall comfort. To our best knowledge, this study may be the first to document the incidence of lace loosening during running, a factor that is often overlooked in research of relevant areas. Although statistical significance was not reached, the absence of lace loosening cases in DLS is noteworthy and likely reflects a lower loosening propensity with the lacing system in the target population. If this trend represented a substantial difference between the two lacing conditions, DLS may be associated with improved perception of foot-shoe stability and subsequent comfort as proposed by Fiedler et al. (8). Further investigation is warranted for more conclusive statements.

We speculated that the enhanced comfort and perceived control are largely attributable to the more consistent and evenly distributed in-shoe pressures across strides afforded by DLS. Prior studies have demonstrated that the dorsal-foot pressure is directly related to perceived comfort and stability of the foot–shoe interface, with tighter or higher-eyelet lacing reducing unwanted foot movement and improving midfoot containment (4, 8, 9, 24). These converging findings underscore the relevance of pressure measures in determining runners' comfort and stability perceptions.

The dorsal foot—particularly the instep—is widely regarded as a primary region of interest when evaluating foot–shoe stability and overall fit (15, 25). Previous studies have documented the peak dorsal pressures and dorsal pressure contact areas under different lacing conditions (9, 26), assuming that both metrics are direct indicators of comfort perception. Although measurable changes in peak dorsal foot pressure have been reported in wrapping closure systems, most studies—including the current one—have found no significant reductions in the peak pressure reading. Findings on dorsal contact areas are also inconsistent (26). Nevertheless, we want to highlight that metrics of the maximal pressure on the dorsal foot may not be the most pertinent variables that underlie the changes in perceived comfort. We found that the normal pressure range of the dorsal foot during running is far lower than the thresholds that trigger perceivable discomfort. In other words, modest differences in peak pressure between lacing systems are unlikely to drive comfort ratings. Instead, some participants of our studies reflected that the larger reduction in wearing comfort with CL was attributed to inconsistent lockdown during stance-swing transitions and subsequently a sensation of “instability.” In light of the scenario, we explored a novel metric—pressure fluctuation within the gait cycle—as a potential indicator of lockdown consistency (12). Our results were in accordance with previous reports. A wrapping closure system such as DLS produces smaller deviations in the dorsal foot pressures within a gait cycle compared to CL, which, as proposed by Honert et al. (11), indicates more even pressure distribution and contributes to consistently improved comfort in running footwear. Based on the manufacturer's specification and testing results, the friction coefficient of the DLS wire is 0.26, which is greatly lower than that of the CL supplied with test shoes. In addition, the DLS wire is thinner and yields a relatively smaller surface contact (Figure 1A). Our conjecture is that, compared to CL, DLS wires allow smoother sliding through the eyelets. This feature facilitates dynamic adjustment of the lacing tension across different tongue regions and distributes the dorsal foot pressure more uniformly within a gait cycle (13).

Similar to other wrapping closure systems, DLS exhibited the biomechanical features of a better foot-shoe fitting alongside with wearer-preferred comfort over CL (11). Measures of EMG in the current study provide additional evidence that indirectly support reduced foot displacement within the shoes in the DLS condition. Notably, tibialis anterior activation was significantly lower during running with DLS than with CL. Given that this muscle is most active at the pre-landing phase to dorsiflex the ankle and increase foot–ground clearance (27, 28), the higher activation observed in the CL condition likely reflects an adaptive response to reduced foot-shoe fitting as additional in-shoe displacement requires a larger foot excursion to lift the shoe and muscular preparation for stable ground contact. However, this interpretation remains inferential without direct measures of in-shoe foot displacement in the present study. Despite this localized EMG difference, no significant alterations were observed in the activation patterns of other major lower limb muscles, nor in joint kinematics or intersegmental coordination variabilities. The results are consistent with previous observations that modified closure-system stability mainly influences local foot mechanics while exerting minimal impact on the global kinematic chain (11). The human motor system is highly redundant, meaning that muscles can be reprogrammed to yield different activation combinations that retain the global gait patterns and motion variabilities (29). Muscle activation is generally more sensitive than joint-level kinematics in responses to small perturbations at distal body segments. When the foot–shoe coupling is slightly disturbed by altered lacing conditions, the motor system can compensate locally—primarily through adjustments in specific muscles such as the tibialis anterior—without requiring large changes in whole-body kinematics. Such muscular adjustments may be associated with an altered energetic strategy and fatigue-related injury projection.

Prior work suggested that lacing systems designed to better wrap the foot can improve running economy without eliciting pain or musculoskeletal pathology (10). Excessive foot movements due to poor foot-shoe compartment, e.g., vertical slipping, were found to modify the runners' landing patterns (25), which could in turn alter joint loading profiles and energy expenditure (30, 31). Looser lacing patterns also increase horizontal foot motion within the shoes (9), which not only compromises foot–shoe coupling but also elevates the energetic cost of locomotion associated with extra muscular effort to stabilize the foot-shoe complex. Tighter fixation, on the other hand, was reported to reduce energy loss and improve performance (13). In the context of injury risk, loosened or unstable lacing has been linked to increased in-shoe foot displacement and heel slippage, conditions that may predispose runners to overuse injuries through repetitive micro-adjustments and altered loading trajectories (9, 24). We therefore propose further investigations to compare the metabolic demands and injury risks between DLS and CL to demonstrate the broader benefits of the wrapping closure system.

Several limitations should be acknowledged when interpreting the findings of this study. First, all measurements were conducted in a controlled laboratory environment, which does not fully capture the variability encountered in real-world trail running. Such environmental variability may amplify the biomechanical differences observed between lacing systems. Second, the present study lacked a formal sample size calculation, which may limit the detection of small effects or low-incidence outcomes. In addition, participants in this study were restricted to a specific age range. The applicability of our findings to other age groups was unclear. Third, the pressure sensor used in this study measured only the total pressure applied to the sensing region. Detailed pressure mapping of more extensive areas of the dorsal foot may reveal spatial distribution patterns that further distinguish lacing performance. Placement of the pressure pad and kinesiology tapes may affect subjective comfort ratings compared with natural footwear conditions. Resolving this technical constraint would require new advances in sensing technology. Fourth, EMG amplitudes were normalized to the maximal activation observed during the running trials rather than measures of maximum isometric muscle contraction. Although this approach is appropriate for studies of within-subject design, it limits the interpretability of the findings on absolute muscle activation levels. Additionally, neither participants nor investigators were blinded to footwear conditions, which possibly introduced bias in comfort ratings/self-selected lacing tightness and confounded the results. Finally, the study examined only the immediate effects of each lacing system, leaving unknown their impacts during prolonged running and the potential long-term adaptations that may emerge through extended use.

## 5 Conclusion

The current study compared the performance of a wrapping closure system and conventional laces in influencing wearing comfort, lock-in stability, running kinematics, and lower-limb muscle activation during prolonged running. The wrapping closure system reduced donning/doffing time and received superior VAS ratings of comfort compared to conventional laces. The dorsal foot pressure fluctuations within a gait cycle and peak activation of the tibialis anterior were lower in wrapping closure than in conventional laces. These findings highlight the potential advantages of a wrapping-closure design in maintaining stable lacing tension on the dorsal foot and the foot-shoe coupling during highly repetitive exercises, which likely leads to better perceived comfort and reduced muscular demand for stabilizing the foot-shoe complex.

## Data Availability

The raw data supporting the conclusions of this article will be made available by the authors, without undue reservation.
